# An actionable *KCNH2* Long QT Syndrome variant detected by sequence and haplotype analysis in a population research cohort

**DOI:** 10.1038/s41598-019-47436-6

**Published:** 2019-07-29

**Authors:** Shona M. Kerr, Lucija Klaric, Mihail Halachev, Caroline Hayward, Thibaud S. Boutin, Alison M. Meynert, Colin A. Semple, Annukka M. Tuiskula, Heikki Swan, Javier Santoyo-Lopez, Veronique Vitart, Chris Haley, John Dean, Zosia Miedzybrodzka, Timothy J. Aitman, James F. Wilson

**Affiliations:** 1 MRC Human Genetics Unit, University of Edinburgh, Institute of Genetics and Molecular Medicine, Western General Hospital, Crewe Road, Edinburgh, EH4 2XU UK; 2 Laboratory of Genetics, HUSLAB, Helsinki University Hospital, Helsinki, Finland; 3 Department of Medicine, University of Helsinki and Helsinki University Hospital, Helsinki, Finland; 4 Heart and Lung Center, Helsinki University Hospital and University of Helsinki, Helsinki, Finland; 5 Edinburgh Genomics, The Roslin Institute and R(D)SVS, University of Edinburgh, Easter Bush, Edinburgh EH25 9RG UK; 6 Medical Genetics Group, University of Aberdeen, Polwarth Building, Aberdeen, AB25 2ZD UK; 7 Department of Medical Genetics, Ashgrove House, NHS Grampian, Aberdeen, AB25 2ZA UK; 8 Centre for Genomic and Experimental Medicine, University of Edinburgh, Institute of Genetics and Molecular Medicine, Western General Hospital, Crewe Road, Edinburgh, EH4 2XU UK; 9 Centre for Global Health Research, Usher Institute of Population Health Sciences and Informatics, University of Edinburgh, Teviot Place, Edinburgh EH8 9AG UK

**Keywords:** Heritable quantitative trait, Medical genetics

## Abstract

The Viking Health Study Shetland is a population-based research cohort of 2,122 volunteer participants with ancestry from the Shetland Isles in northern Scotland. The high kinship and detailed phenotype data support a range of approaches for associating rare genetic variants, enriched in this isolate population, with quantitative traits and diseases. As an exemplar, the c.1750G > A; p.Gly584Ser variant within the coding sequence of the *KCNH2* gene implicated in Long QT Syndrome (LQTS), which occurred once in 500 whole genome sequences from this population, was investigated. Targeted sequencing of the *KCNH2* gene in family members of the initial participant confirmed the presence of the sequence variant and identified two further members of the same family pedigree who shared the variant. Investigation of these three related participants for whom single nucleotide polymorphism (SNP) array genotypes were available allowed a unique shared haplotype of 1.22 Mb to be defined around this locus. Searching across the full cohort for this haplotype uncovered two additional apparently unrelated individuals with no known genealogical connection to the original kindred. All five participants with the defined haplotype were shown to share the rare variant by targeted Sanger sequencing. If this result were verified in a healthcare setting, it would be considered clinically actionable, and has been actioned in relatives ascertained independently through clinical presentation. The General Practitioners of four study participants with the rare variant were alerted to the research findings by letters outlining the phenotype (prolonged electrocardiographic QTc interval). A lack of detectable haplotype sharing between c.1750G > A; p.Gly584Ser chromosomes from previously reported individuals from Finland and those in this study from Shetland suggests that this mutation has arisen more than once in human history. This study showcases the potential value of isolate population-based research resources for genomic medicine. It also illustrates some challenges around communication of actionable findings in research participants in this context.

## Introduction

The Northern Isles of Scotland (Orkney and Shetland) have been isolated from the rest of the British Isles by their extreme northern geographic position and this isolation is reflected in substantial population genetic structuring both within and between these archipelagos and mainland Britain^1–4^. The Orkney Complex Disease Study (ORCADES) began in 2005 in the Orkney Islands and consists of a rich resource of more than 2,000 deeply phenotyped subjects^5^. Over 2,000 volunteers from the archipelago of Shetland were recruited to the Viking Health Study Shetland in 2013–2015. All of these participants, collectively termed “VIKING”, have at least two grandparents from the Northern Isles, and more than 90% have three or four such grandparents. There is a high degree of kinship^6^, evidenced both in the pedigrees and in genome-wide genotype data which are available for all 4,300 participants in the VIKING cohort. Furthermore, linkage to National Health Service (NHS) routine electronic health record (EHR) data adds a longitudinal component to the study through clinical measures and outcomes. The populations of Orkney and Shetland have a number of characteristics, including increased genetic^7–9^ and environmental homogeneity, which are highly favourable for the identification of genes influencing quantitative traits and risks of disease^10^. We have shown previously that isolate populations are enriched for homozygous loss of function variants of low frequency^11^, because rarer variants are relatively more likely to be brought into the homozygous state within the long runs of homozygosity present in these populations^5,12^.

The genetic drift in isolated populations leads to an increased frequency of some otherwise rare variants, which is potentially useful in rare variant association studies^13^. Most rare variants that have an important role in disease today arose during approximately the last 100 generations, and provide signatures of population history^14^. There is considerable evidence that some variants of low frequency have much larger effects on biomedical traits than is usual for more common variants (reviewed in^15^), and it is rare variants of large effect that are of particular clinical relevance. Availability of whole genome sequence data for a subset of VIKING study participants facilitates identification and investigation of such variants and builds on our earlier research demonstrating enrichment of rare and low frequency functional variants in isolated populations^16^.

During the recruitment of volunteers to the VIKING Study, one participant offered the research team a letter s/he had received from the local regional genetic service which informed him/her of a familial risk of long QT syndrome and detailed the causative actionable variant. This is c.1750G > A; p.Gly584Ser in the *KCNH2* gene, which encodes a potassium channel in which this variant causes abnormal inactivation gating^17^. This participant is part of a large pedigree of more than 30 individuals within the VIKING research cohort, all of whom had SNP array genotypes and 10 of whom had whole genome sequencing performed on their DNA. These data facilitated genomic analyses, allowing determination of whether any participants with this actionable variant were present in the entire VIKING cohort.

## Methods

### Recruitment and DNA extraction

Recruitment to the Viking Health Study Shetland took place from 2013–2015. Selection criteria for the volunteer participants were age over 18 years and two or more grandparents born in the Shetland Isles in the north of Scotland. More than 90% had three or four grandparents from Shetland and most were related individuals from large kindreds. The participants attended two clinics, one for fasting venepuncture and one for physical measurements, and provided broad-ranging consent for research, including for whole genome sequencing (WGS), analysis of rare variants and for their research data to be linkable to their NHS electronic medical records. Prior to quality control exclusions, the genome-wide SNP genotyped set comprised 2,122 participants. Blood (or very occasionally saliva) samples from participants were collected, processed and stored using standard operating procedures and managed through a laboratory information management system at the Edinburgh Clinical Research Facility, University of Edinburgh. A biobank of plasma, serum, whole blood and urine is available.

### Genotyping

DNA from all VIKING participants was quantitated using picogreen and diluted to 50 ng/μL; 4 μL were then used for genome-wide genotyping on the HumanOmniExpressExome-8 v1.2 BeadChip (Illumina), with Infinium chemistry^18^. DNA samples from two c.1750G > A; p.Gly584Ser LQTS patients from Finland^17,19^ were quantitated and genotyped using v1.6 of the same Illumina chip, at the same core facility. The genotyping of these samples was done on a single chip alongside repeat genotypes of two sequenced Shetland samples, as positive and negative controls for the Shetland haplotype containing the rare actionable variant in *KCNH2*. Genotyping quality control for the VIKING cohort was performed as follows: individuals with a call rate less than 98% were removed, as were SNPs with a call rate less than 98%, or Hardy-Weinberg equilibrium *p-*value less than 10^−6^. Mendelian errors, determined using relationships recorded in the pedigree, were removed by setting the individual-level genotypes at erroneous SNPs to missing. Ancestry outliers (five individuals) who were more than six standard deviations away from the mean for the first two PCs, in a principal component analysis of VIKING combined with individuals from the Yoruba, Japanese and Han Chinese populations in the 1000 Genomes Project^20^, were excluded. A total of 2,011 individuals (843 male and 1,268 female participants) passed all quality control thresholds. The number of genotyped SNPs that passed all quality control parameters was 928,791. Genome-wide identity-by-descent inferred from identity-by-state (using the *–genome* function in PLINK 1.9^21^ (www.cog-genomics.org/plink/1.9/) was used to identify genetic relationships and a number of individuals whose family history and genotype data did not match the pedigree were removed or resolved through other genetic matches.

### DNA sequencing

Selection of the participants for whole genome sequencing (WGS) from within the SNP genotyped cohort of 2,011 participants used the ANCHAP method^22^ to represent most effectively the haplotypes present across the entire sample. Unrelated individuals from the largest families were selected first, followed by those from smaller families, until eventually related individuals were selected to best represent the variation in the full cohort. 500 DNA samples from VIKING underwent WGS at Edinburgh Genomics, University of Edinburgh. PCR-free paired end WGS (TruSeq DNA PCR-Free, Illumina) was run on a HiSeqX platform. The average fold coverage was greater than 35 (range 27.07–63.53).

An Edinburgh Genomics bioinformatics pipeline was applied to the data and involved removing adapter sequences, removing duplicates, alignment and base recalibration. The Bioinformatics Analysis Core at the Institute of Genetics and Molecular Medicine used the provided intermediate genomic variant call files (gVCFs) to produce high-quality variant call files (VCFs) for the downstream analyses. This used the Genome Analysis Toolkit (GATK)^23^ HaplotypeCaller, the hg38 human genome reference assembly (including alt, decoy and HLA sequences) and followed GATK Best Practices. Overall concordance between array and WGS-derived genotypes was evaluated with the GATK Genotype Concordance tool and was found to be 99.6%. All 500 WGS datasets were retained for further analysis.

The rs199473428 SNP (*KCNH2* c.1750G > A; p.Gly584Ser) genotypes were extracted from the VCFs. In the one occurrence of the minor allele, the call was of good quality, with the site covered by 47 reads (23 REF, 24 ALT). The variant was also analysed by targeted Sanger sequencing of PCR products amplified from selected genomic DNA samples. The primers KCNH2_F (5′CGTGCTGTTCTTGCTCATGT3′) and KCNH2_R (5′TAGAGCGCCGTCACATACTT3′) were designed using Primer3 software^24^ and used to generate a fragment of 204 base pairs for analysis.

### Pedigree information

Records of the births, marriages and deaths in Orkney and Shetland are kept at the General Register Office for Scotland (New Register House, Edinburgh). These records, along with relationship information obtained from study participants and genealogies available online, were used to assemble a large (>40,000 person) pedigree for participants in our studies from Orkney and Shetland. This pedigree was corrected to reflect the genetic kinship between individuals, using the merged Orkney and Shetland genotype data.

### Haplotype analysis

The array SNP genotyped data were phased using Shapeit2 v2r837^25^, with the duoHMM option that takes advantage of the family-based nature of the data^26^. Then, the phased genotype data were used to determine a shared haplotype around the rs199473428 variant using coarse and fine methods, all performed using R 3.3.0^27^. Haplotypes were first defined as all SNPs in windows of 0.2 Mb increments surrounding the unmeasured SNP of interest (coarse method). We defined the carrier haplotype at the given window size by selecting the haplotype where the genotypes (coded as 1 or 0, reflecting having a reference or alternative allele) of the initial three positive samples matched, and searched for the same haplotype in the remainder of the 2,011 samples.

A single variant-based haplotype search was performed to determine the haplotype length between the original carrier and the candidates from the second family using a stepwise approach (fine method). Starting from four variants that were physically the closest to the unmeasured rare variant, one SNP variant at a time was added to define a haplotype. The procedure was repeated until haplotypes of two individuals (known carrier and candidate carrier) no longer matched, providing variant-level resolution of the haplotype length.

### Identity-by-descent (IBD)

Regional IBD sharing between pairs of individuals were assessed using version 2.1.6 of the KING (Kinship-based Inference for GWAS) toolset^28^. Genome-wide genotypes (~550,000 non-monomorphic common SNPs) in PLINK format^21^ were used as input to assess sharing of two, one, or no haplotypes identical-by-descent along each autosome.

### Electrocardiogram (ECG) phenotyping

As part of the measurement clinic, electrocardiograms were recorded for all participants using a Universal 12-Lead Interpretive ECG system (Numed Healthcare). Subjects were kept supine on an examination couch and an electronic 30 second ECG recorded using Cardioview software and a standard operating procedure. The QT is the time between the start of the Q wave and the end of the T wave. QT intervals were corrected for heart rate (QTc) using Bazett’s formula.

### Return of results

A process for how the actionable finding should be communicated, using a mechanism in line with the favourable Research Ethics Committee (REC) opinion given to the VIKING cohort and the Medical Research Council (MRC) Framework on the feedback of health-related findings in research, was reviewed and approved by the NHS South East Scotland Research Ethics Committee (Amendment number: 12/SS/0151/AM05 SA03), the NHS Shetland R&D Office and the Ethics Advisory Group of the Scottish Genomes Partnership. Letters were sent using recorded delivery to the General Practitioners (GPs) of four participants, together with a copy of the electrocardiogram measured in the recruitment clinic. The letter to the GPs of the selected participants is in line with the consent participants gave upon recruitment to the study.

### Ethics approval and consent to participate

Eligible participants (greater than 18 years of age and with two or more grandparents from Shetland) were recruited to the Viking Health Study Shetland, REC reference: 12/SS/0151 (South East Scotland Research Ethics Committee, NHS Lothian). VIKING participants gave written informed consent for research procedures including electronic health record linkage, with NHS datasets accessed using a process essentially as described for the Generation Scotland cohort^29^. The data linkage and access to NHS Scotland-originated data was approved by the Public Benefit and Privacy Panel for Health and Social Care (Ref 1718-0380). All methods were performed in accordance with the relevant guidelines.

## Results

### Detection of a LQTS rare variant

**Figure 1:**
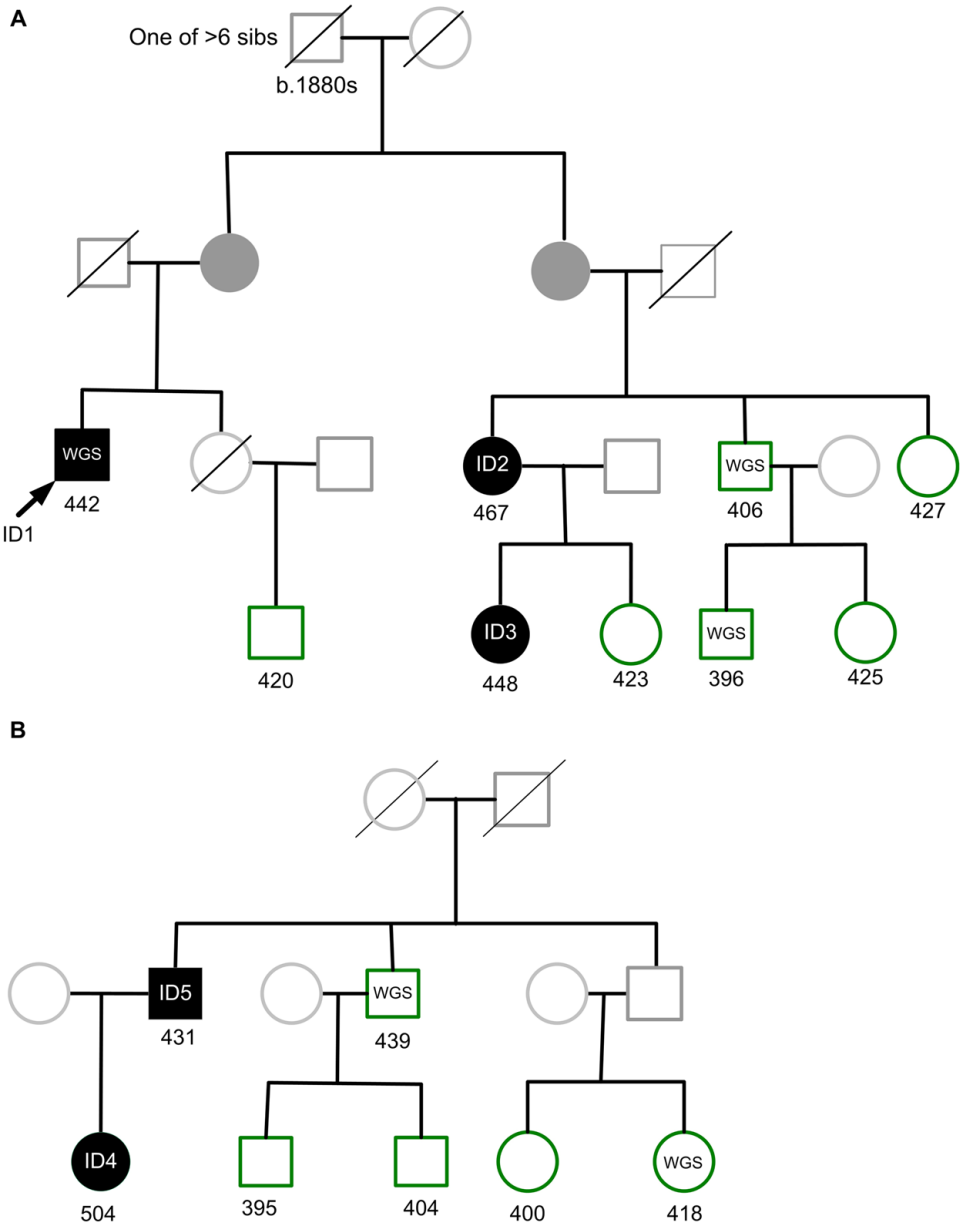
Extracts from two multi-generational VIKING pedigrees. (**A**) Excerpt from a pedigree containing more than 30 genotyped participants, Family A. The initial participant with WGS (ID1) is indicated by an arrow and his two carrier relatives (ID2 and ID3) in the pedigree are shaded in black. (**B**) Excerpt from a second pedigree (Family B) containing 10 genotyped participants. The two participants identified by haplotype analysis, ID4 and ID5, are shaded in black. Other genotyped VIKING participants in each family are shown in green, and participants with WGS data are indicated. Family members not in the research cohort are represented in lighter shading. There are many other family members not shown. Obligate carriers of the rare variant are filled in dark grey. Values below each individual are their measured QTc interval.

500 whole genomes of VIKING participants were sequenced to high depth (an average fold coverage per sample of >35). These WGS data add to the genome-wide genotype and deep phenotype data, which are available on this research cohort of more than 2,000 people with Shetlandic ancestry. Inspection of variant call files (VCFs) showed (with high confidence) that one of the sequenced participants (a distant relative of the person who provided the NHS letter) carries the rare pathogenic coding variant c.1750G > A; p.Gly584Ser in *KCNH2* (NM_000238.3), rs199473428. This missense mutation is classified as pathogenic/likely pathogenic in the ClinVar archive of human genetic variants^30^, variation ID 67261. It has been reported in multiple individuals to segregate with long QT syndrome (LQTS), including in large multigenerational kindreds^17^. The result (presence of the variant) in the single research participant from the WGS analysis was confirmed by Sanger sequencing (Methods). DNA samples from all members of the pedigree (“pedigree A”) of the c.1750G > A; p.Gly584Ser participant identified by WGS were Sanger sequenced, and two relatives were found to carry the same variant (Fig. 1). All three have a somewhat prolonged QTc interval on ECGs taken in the recruitment clinic (Table 1).

**Table 1:** Characteristics of the rs199473428 rare variant carrier participants and their method of identification.

Participant	Gender	Age Range at Clinic (years)	QTc (ms)	rs199473428 Identification Method	Letter to GP
ID1	M	46–50	442	WGS, confirmed by Sanger sequencing	Yes
ID2	F	46–50	467	Sanger sequencing of pedigree of ID1	Yes
ID3	F	21–25	448	Sanger sequencing of pedigree of ID1	Yes
ID4	F	36–40	504	Haplotype, confirmed by Sanger sequencing	Yes
ID5	M	66–70	431	Haplotype, confirmed by Sanger sequencing	No

QTc values were measured in the research clinic (Methods).

### Definition of a carrier haplotype

**Figure 2:**
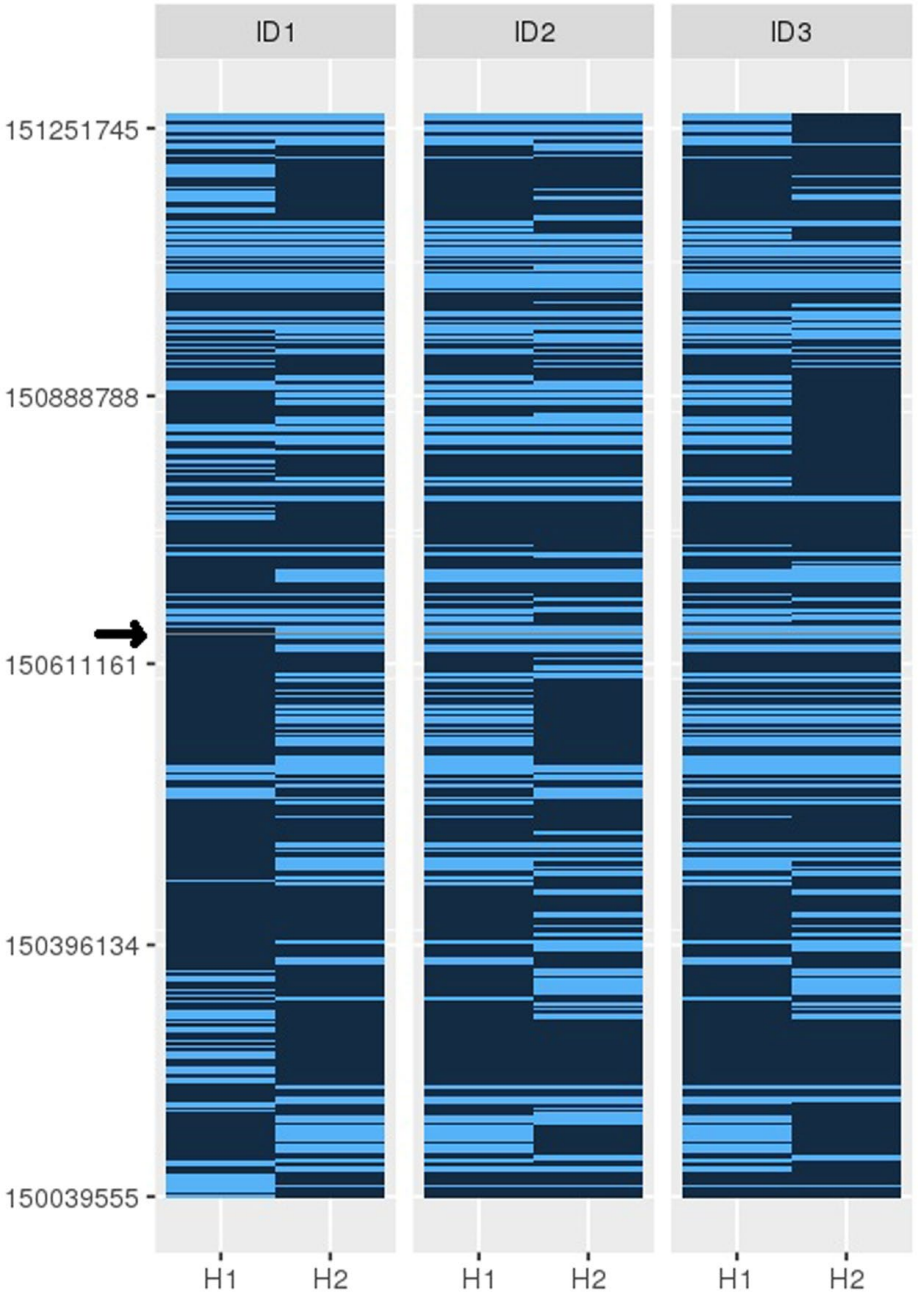
Analysis of shared haplotypes using phased genotypes. The figure shows the genomic positions of informative SNPs in a 1.22 Mb region of Chromosome 7, with the rs199473428 SNP indicated by a grey line (arrowed). Haplotypes (H1 and H2) of the participant with WGS data (ID1) and his two relatives (ID2 and ID3) in Family A are shown. The carrier haplotype was identified as H2 of ID1, and H1 of individuals ID2 and ID3. The extent of sharing goes beyond the 1.22 Mb window shown.

Even in the VIKING cohort where detailed genealogical data are available, relationships between some pairs of individuals are likely to be unrecorded. While their overall genomic sharing may be low, these unrecorded relative pairs can be informative for a range of genetic studies^31,32^, as they may share relatively long genomic segments inherited identical-by-descent from a shared ancestor. The exemplar SNP is too rare to be present in imputed data derived using the Haplotype Reference Consortium (HRC)^33^, but the more common directly genotyped SNPs surrounding it were used to estimate haplotypes in the VIKING (Shetland) cohort (Methods). Haplotypes were defined using the three positive samples (the WGS “proband” and his two relatives) from the kindred. As these are close relatives (Fig. 1A), the extent of genome sharing is anticipated to be high. We therefore began with an initially coarse search, defining haplotypes using 0.2 Mb incremental windows surrounding the SNP of interest (rs199473428; UCSC 7-150648731-C-T, GRCh37). Haplotypes from the phased genotype data for the three known carriers were then compared, to define those which are common to all (Fig. 2).

The 0.2 Mb incrementally longer haplotypes were then sought in all 2,011 genotyped participants in the VIKING cohort. In addition to the three known carriers, three unrelated individuals shared 0.42 Mb, ten shared 0.82 Mb and two participants shared 1.22 Mb long haplotypes around the variant, shown in Fig. 2. Out of the 13 individuals sharing shorter haplotypes of 0.82 Mb or less, three had been whole-genome sequenced and three members of Family A sequenced for the *KCNH2* mutation by targeted (Sanger) sequencing. This confirmed that all of these six participants are *KCNH2* c.1750G > A; p.Gly584Ser non-carriers, and therefore that the shorter haplotypes are not sufficiently unique to indicate carrier status.

In contrast, the two individuals sharing the 1.22 Mb long haplotype (father and daughter) were found to have the *KCNH2* c.1750G > A; p.Gly584Ser variant, using Sanger sequencing. The two new individuals in “Pedigree B” (father and daughter, ID5 and ID4, respectively, Table 1 and Fig. 1B), are not closely related to the other three by conventional analyses of genotype using PLINK (average genome-wide IBD sharing less than 5%, i.e. PiHAT < 0.05). They do not share known pedigree ancestors with the original family back to *c*1770, but do share the same long (1.22 Mb) haplotype. One of these two new individuals, ID4, had a phenotype of a prolonged QTc interval (Table 1) at recruitment. Phenotype and genotype relationships in LQTS patients are complex, but mutation carriers have a higher risk of cardiac events than unaffected family members, even in the absence of QTc prolongation^34^.

**Figure 3:**
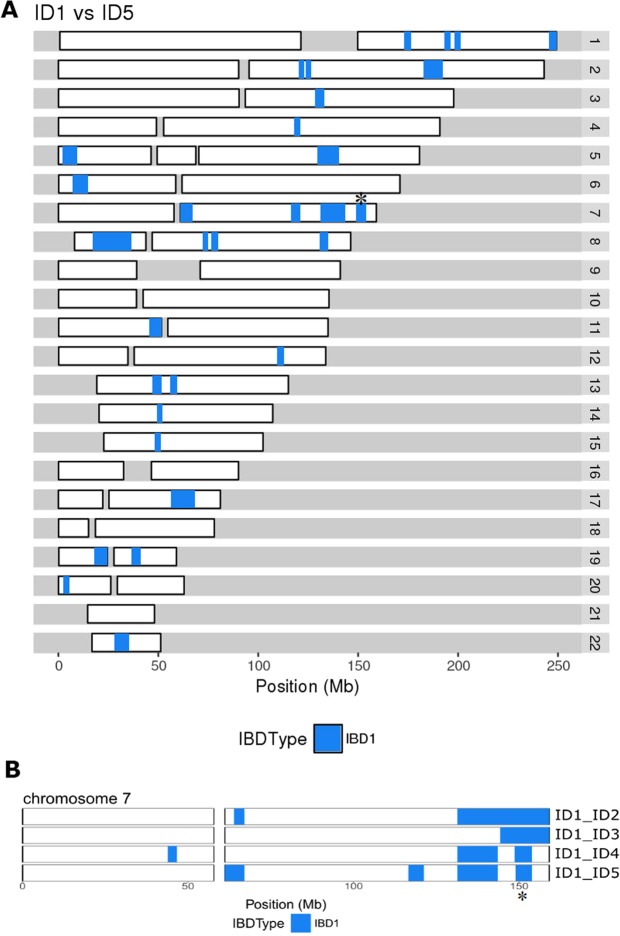
(**A**) Genomic sharing between individuals ID1 in Family A and ID5 in Family B. Chromosomes (autosomes) 1 to 22 are shown horizontally, with the scale in megabases (Mb). (**B**) Comparison of the genomic sharing on chromosome 7 between ID1 and each of the other four rare variant heterozygotes. The haplotype has been broken down by recombination, comparing kindred A (ID1, ID2, ID3) and kindred B (ID4, ID5). In both parts of the Figure, segments of identity-by-descent (IBD1) are indicated in blue. The 4.93 Mb segment shared by all five heterozygotes on chromosome 7q, that includes the *KCNH2* locus at 150 Mb, is marked with a *. Regions with no identity-by-descent (IBD0) are in white and centromere regions are shown as gaps.

Identification of the second carrier family B (Fig. 1B) enabled more detailed, fine-level analysis of the extent of the shared haplotype. The coarse 0.2 Mb incremental symmetrical analysis was followed by single variant-based analysis, to enable more precise definition of haplotype length (Methods). With this variant-based haplotype search, the haplotype shared between the two families A and B was found to be 4.93 Mb long. This is illustrated schematically for chromosome 7 in Fig. 3B.

Analysis of identity-by-descent (IBD) between members of each of the two families A and B revealed a pattern of sharing of long genomic segments throughout the genome, as illustrated in Fig. 3A. The long shared segment (4.93 Mb) that is IBD around *KCNH2* on Chr7 is indicated (Fig. 3). The overall genomic sharing between participants ID1 and ID5 assessed with KING [27] totals 105 Mb in 11 segments (over 5 Mb), the longest being 19.1 Mb in length (Fig. 3A). Moreover, a further 20 segments above 2.5 Mb in length, totalling 73 Mb, were shared. Comparison with 29 third cousin pairs from the extended pedigree of kindred A shows an average genomic sharing of 82 Mb in 8 segments over 5 Mb, the longest being 23 Mb in length, with a further 17 segments above 2.5 Mb in length, totalling 61 Mb.

Thus the sharing we observe is compatible with a relationship of approximately third cousins (i.e. sharing a set of great-great-grandparents) between the two individuals ID1 and ID5. While the variance around expectations of sharing is very high and an accumulation of deeper relationships likely contributes to the kinship, the simplest explanation is a previously unknown relationship between these two kindreds about four generations ago. The incidence of misattributed genetic relationships is generally not well known, particularly historically, but the cumulative nature over the generations suggests that such effects will be of significant importance in the segregation of actionable variants throughout a population.

### Frequency of the mutation in other populations

The c.1750G > A; p.Gly584Ser variant was first described in LQTS patients in Finland^35^, one of eight molecularly-defined mutations in *KCNH2* in 39 Finnish LQTS patients^19^. However, this variant is not one of the four most common potassium channel mutations which account for a large proportion of LQTS cases in Finland^36^. In order to assess whether the c.1750G > A; p.Gly584Ser mutation observed in Finland^19,35^ arose on the same haplotype background as that in Shetland, we genotyped two independent Finnish samples (including one from the family in^17^). Analysis of haplotype sharing reveals that neither of the Finnish samples shares the same haplotype as the Shetland families, and indeed the Finnish samples do not share a haplotype with each other across the region of interest. Hence it is most likely that the mutation has arisen at least three separate times. This variant has also been reported in LQTS patients ascertained in clinics from North America and Europe^37,38^, although their ancestral geographic origins were not clearly defined. The p.Gly584Ser change is a non-conservative amino acid substitution in the pore domain of the potassium channel protein encoded by *KCNH2*, and has a dominant (or additive) pattern of inheritance.

The c.1750G > A; p.Gly584Ser variant is sufficiently rare that it is not observed at significant frequency in large cosmopolitan population cohorts^30^. We found that in ORCADES (including > 2000 Orcadians, who are genetically closest to Shetlanders), no participants were observed with the defining long (1.22 Mb) haplotype. The Genome Aggregation Database (gnomAD v2.1) of 123,136 exomes and 15,496 genomes from unrelated individuals sequenced as part of various disease-specific and population genetic studies^39^ has two instances of the variant (in European Non-Finnish populations), an allele frequency of 8.1 × 10^−6^. The frequency in Shetland (5 in 2,011 genomes, ~0.0012, although this counts multiple alleles in a family) is thus ~150-fold increased over that in non-Finnish Europeans, emphasising the strength of genetic drift on ultra-rare variants in this population isolate. It seems likely that this instance is an ancestral Shetlandic variant, on a rare Northern European haplotype.

### Communication of results

LQTS is a familial cardiovascular disorder characterised by prolongation of the QT interval on ECG and risk of sudden death. Table 1 shows some characteristics of the five VIKING participants heterozygous for the *KCNH2* rare variant. Inspection of electronic health record (EHR) data (SMR00 out-patients database) indicated that neither of the two participants identified by haplotype had any linked entry corresponding to the speciality of clinical genetics, whereas two of the three initial related family members did. The third initial family member is likely to have been identified by cascade testing, although this was not recorded in the EHR as an out-patients attendance. A check of the EHR National Records of Scotland Deaths dataset indicated that all five p.Gly584Ser participants were alive in the most recent data release (December 2017, six months before the GPs were contacted, below).

In the recruitment protocol, participants consented that “a copy of the results of the commonly used medical tests and information from the replies to the questionnaire (for example on smoking) may be sent to my GP” and “I agree that my GP may be contacted if new research findings suggest that I might need more tests”. A process was established for how the actionable finding should be communicated, using a mechanism in line with the favourable REC opinion given to the VIKING cohort and the MRC Framework on the feedback of health-related findings in research. The General Practitioners of four of the five carriers (those with prolonged QTc interval from ECG measurement, Table 1) were alerted to the research findings by letter, describing the phenotype, enclosing a copy of the ECG measured in the recruitment clinic and providing contact details for further information.

It was possible to link the whole genome sequenced VIKING research participant in Family A to a large Shetland family which had been previously investigated by the North of Scotland NHS Genetics Service, as this individual had reported participation in the study at attendance for cascade testing. To date 169 relatives from this family have enquired about cascade testing, of whom 158 have had NHS genetic testing for the variant. Only 28 are heterozygous for the variant (many distant relatives were contacted by a family member and advised to seek cascade testing despite no connecting individuals being available). The mean QTc amongst the heterozygotes was 456 ms (range 415–530 ms), whereas for those without the variant, the mean QTc was 413 ms (range 362–451 ms). This illustrates prolongation of the QTc interval in carriers of the G584S variant, as previously described by others, e.g.^17^. Three heterozygotes have been symptomatic: one had a sudden death before the age of 50, another required an implantable defibrillator for ventricular arrhythmia before the age of 65, while the third had a syncope and seizure of presumed cardiac origin as a teenager. The twenty-five asymptomatic heterozygotes have a mean age of 46 (range 3 months–91 years). The variant therefore seems generally of low penetrance, although it has the potential to be associated with fatal arrhythmia in the absence of appropriate clinical management.

## Discussion

Family-based studies, especially those utilising population isolates, are enriched for rare variants because these may, by chance, be passed on from founders to many descendants within a family, a form of genetic drift^40^. When the same mutation is found in different families, this implies either a common ancestor (founder), or multiple *de novo* mutations. The haplotype analyses presented here have defined a rare long haplotype, on which the original mutation may have occurred in a Shetland founder individual. This approach identified two individuals who would not have been ascertained by genealogy or cascade testing of relatives from the initial pedigree, but for whom there is compelling evidence that they carry the ancestral variant. One of the two carriers identified through the haplotype analysis also has a phenotype of a prolonged QTc interval. Penetrance is variable in LQTS, as seen here. Consistent with analyses in other populations, an apparently pathogenic variant occurs in the Shetland Isles cohort at a frequency (1 in 400) which fits poorly with the frequency of the clinical disease. LQTS is no exception, with the population prevalence being reasonably well established at 1 per 2000^41^. The true penetrance of many rare alleles is uncertain, and may be subject to ascertainment bias^42^.

The General Practitioners of the four participants with a prolonged QTc measurement were alerted to the research finding by letter. These letters created the opportunity for each symptomatic individual carrying the variant to undergo genetic counselling and be offered clinical grade genetic testing and, if positive, cardiac evaluation. This would apply if the individual had not already been tested through NHS genetic services. This in turn would provide the opportunity for cascade testing of close relatives of each newly ascertained carrier, through normal NHS procedures. Those found to have the rs199473428 rare variant would also be offered cardiac assessment and appropriate therapy, for example, β-blockade. Risk reduction can also be achieved through lifestyle modifications, avoidance of drugs that prolong the QT interval and where ventricular arrhythmia is documented despite medical therapy, an implantable cardiac defibrillator can be considered^43^.

The American College of Medical Genetics and Genomics (ACMG) has issued detailed guidance on feedback of secondary findings in clinical exome or genome sequencing (i.e. feedback of findings in genes whose analysis was not the primary reason for the sequencing) and this includes the LQTS gene *KCNH2*^44^. The guidance indicates an expectation that variants either known or expected to be pathogenic will be reported back, but the proposal for such secondary analysis has raised controversy. As part of the governance process of the UK 100,000 genomes project, a detailed review of which variants in which genes should be fed back to consenting research participants as additional findings is being discussed^45^. In population cohort studies where the governance and ethics approvals allow return of actionable genomic results, this would seem an appropriate list for genomics research projects to consider using, as it will only include genes where there is a strong likelihood that variants which affect gene function will have a clinical impact. However, the known or expected pathogenicity (and therefore actionability) of any specific variant to be returned to participants should ideally first be reviewed with the local NHS multi-disciplinary team. A range of rates and types of clinically-relevant genetic findings have been reported in different population cohorts^46^, the largest study of which to date (50,000 people) gave a figure of 3.5% of individuals harbouring deleterious variants in 76 clinically actionable genes^47^.

The “exemplar” presented here illustrates some of the challenges around communication of actionable research findings to participants, a topic attracting considerable attention^48,49^. For example, it has been suggested that a translational research collaboration could be “built onto well-characterized populations with already available sequence data (in a biobank/research environment), risk factor information, intervention information, and clinical outcomes”^50^. The VIKING cohort described here could be one such population. A major challenge is that previously clear lines of demarcation between “research” and “clinical practice” may be starting to blur, as whole genome sequencing at scale becomes commonplace (it has been predicted that more than 60 million people will have their genomes sequenced in a healthcare context by 2025)^51^ and very large population research cohorts with genetic data, exemplified by the 500,000 research volunteers in the UK Biobank^52^, become the norm. However, many such cohorts, including the UK Biobank, explicitly informed participants at recruitment that there would be no feedback of genetic data. This approach may prove to be increasingly unpopular as the existence of detailed research sequence data becomes more widely known to the participants, and its clinical value to a significant percentage of individuals becomes increasingly apparent. In recognition of this, new research cohorts such as the US National Institutes of Health “All of Us Research Program” plan to provide participants with access to results, including genetic/genomic information, according to their preferences^53^. However, this also raises a range of challenges, both logistical and ethico-legal, including potential harm that might befall participants or members of their family as a result of data return^48^. Furthermore, although asking participants for new consent on return of results is an option, we^54^ and others^55^ have demonstrated some of the difficulties of relying upon consent as a means of ensuring that changing data access practices are rendered ethical.

## Conclusions

Here, we have described analyses leading to the identification of five individuals in VIKING carrying a rare actionable genetic variant. Two of these participants could not have been discovered by cascade screening and testing from other carriers in the pedigree known to the NHS. This highlights the potential of cohort studies engaged in genomic medicine to directly benefit participants and complement family-based genetics healthcare services.

## Data Availability

There is neither research ethics committee approval, nor consent from individual participants, to permit open release of the individual level research data underlying this study. The datasets generated and analysed during the current study are therefore not publicly available. Instead, the haplotype data and/or DNA samples are available from the corresponding author Professor Jim Wilson (accessQTL@ed.ac.uk) on reasonable request, following approval by the VIKING Data Access Committee and in line with the consent given by participants.
